# Comparison of Bioelectrical Impedance-Based Methods on Body Composition in Young Patients with Obesity

**DOI:** 10.3390/children8040295

**Published:** 2021-04-11

**Authors:** Alexandra Thajer, Gabriele Skacel, Katharina Truschner, Anselm Jorda, Martin Vasek, Brian Horsak, Johanna Strempfl, Alexandra Kautzky-Willer, Franz Kainberger, Susanne Greber-Platzer

**Affiliations:** 1 Division of Pediatric Pulmonology, Allergology and Endocrinology, Department of Pediatrics and Adolescent Medicine, Medical University of Vienna, Waehringer Guertel 18–20, 1090 Vienna, Austria; gabriele.skacel@akhwien.at (G.S.); kathianna568@gmail.com (K.T.); anselm.jorda@meduniwien.ac.at (A.J.); martin.vasek@meduniwien.ac.at (M.V.); susanne.greber-platzer@meduniwien.ac.at (S.G.-P.); 2 Institute of Health Sciences, St. Pölten University of Applied Sciences, Matthias-Corvinus-Straße 15, 3100 St. Pölten, Austria; brian.horsak@fhstp.ac.at; 3 Department of Physiotherapy, St. Pölten University of Applied Sciences, Matthias-Corvinus-Straße 15, 3100 St. Pölten, Austria; Johanna.Strempfl@fhstp.ac.at; 4 Gender Medicine Unit, Division of Endocrinology and Metabolism, Department of Internal Medicine III, Medical University of Vienna, Waehringer Guertel 18–20, 1090 Vienna, Austria; alexandra.kautzky-willer@meduniwien.ac.at; 5 Division of Neuro- and Musculoskeletal Radiology, Department of Biomedical Imaging and Image-Guided Therapy, Medical University of Vienna, Waehringer Guertel 18–20, 1090 Vienna, Austria; franz.kainberger@meduniwien.ac.at

**Keywords:** pediatric obesity, body composition analysis, bioelectrical impedance analysis, air displacement plethysmography, DXA, body fat percentage, fat mass, fat free mass

## Abstract

(1) Background: The determination of body composition is an important method to investigate patients with obesity and to evaluate the efficacy of individualized medical interventions. Bioelectrical impedance-based methods are non-invasive and widely applied but need to be validated for their use in young patients with obesity. (2) Methods: We compiled data from three independent studies on children and adolescents with obesity, measuring body composition with two bioelectrical impedance-based devices (TANITA and BIACORPUS). For a small patient group, additional data were collected with air displacement plethysmography (BOD POD) and dual-energy X-ray absorptiometry (DXA). (3) Results: Our combined data on 123 patients (age: 6–18 years, body mass index (BMI): 21–59 kg/m²) and the individual studies showed that TANITA and BIACORPUS yield significantly different results on body composition, TANITA overestimating body fat percentage and fat mass relative to BIACORPUS and underestimating fat-free mass (*p* < 0.001 for all three parameters). A Bland–Altman plot indicated little agreement between methods, which produce clinically relevant differences for all three parameters. We detected gender-specific differences with both methods, with body fat percentage being lower (*p* < 0.01) and fat-free mass higher (*p* < 0.001) in males than females. (4) Conclusions: Both bioelectrical impedance-based methods provide significantly different results on body composition in young patients with obesity and thus cannot be used interchangeably, requiring adherence to a specific device for repetitive measurements to ascertain comparability of data.

## 1. Introduction

Obesity is a health issue of world-wide relevance and of increasing prevalence, considered to have reached the level of a pandemic disease over the past 50 years [1]. Besides the often-perceived general reduction of quality of life, obesity is known to profoundly increase the risk for numerous diseases such as diabetes [2], cardiovascular diseases [3], cancer [4], and, as has recently become evident, the severity of viral diseases such as COVID-19 [5,6]. The enhanced prevalence of obesity is already detectable in children and adolescents and a recent study found that 90% of children with obesity at the age of 3 years have overweight at adolescence [7], and almost 60% with obesity at 2 to 19 years were predicted to be obese at an age of 35 [8]. Available longitudinal data suggest that early obesity also enhances the risk of developing metabolic syndrome [9] among numerous other complications [10], and long-term observational studies even identified childhood adiposity as a predictor for morbidity and mortality independent of the weight status in adulthood [11]. The early detection of signs indicating enhanced risk of developing obesity such as unusually high body fat or body fat percentage during childhood and adolescence thus appears of critical importance to allow one to take preventive measures or to initiate medical interventions before the development of obesity-related diseases is triggered.

Among the different methods to diagnose overweight and obesity, the determination of body fat and of total body composition is of particular relevance. Different approaches for correct classification include measurements with bioelectrical impedance analysis (BIA) [12], air displacement plethysmography [13], and dual X-ray absorptiometry (DXA) [14], all of which are reportedly used in clinical practice. DXA is described as a reference method for body composition determination [11,15] but is an expensive tool therefore with limited availability in centers and patients are exposed to radiation. Bioelectrical impedance-based determination of body composition is a relatively simple method, not very time-consuming, non-invasive, and does not expose the patient to radiation [12]. It is widely applied in clinical practice and appears ideally suited for the use with children and adolescents [16]. However, there are different devices available using bioelectrical impedance measuring principles and only limited data on young patients with obesity are available. It is crucial to fill this gap in the literature concerning different bioelectrical impedance devices in children and adolescents with obesity.

Although these methods are well established for the application with adult patients, data on their use in children and adolescents with obesity are associated with considerable uncertainty, since several assumptions underlying these methods may be valid in adults but not in children. For example, changes in protein, water and mineral content occur during growth and maturation and affect the estimates of percent fat and fat-free mass [17]. Additionally, the share of different body compartments is not a constant from childhood to adulthood and shows considerable developmental differences between boys and girls [18]. Observations in 5–7 year old school kids even indicated changes in fat mass and distribution that occur totally independently from changes in body mass index (BMI) [15]. Thus, methods applied to determine body composition in adults need to be validated for their use in children [17,19]. In addition, as different methods may produce variable results, it is important to know how these methods compare with each other to be able to set into perspective data generated using a particular methodology and to evaluate the impact of individualized treatment regimes.

Recently, we could publish differences related to body composition in children with obesity examined by two methods based on bioelectrical impedance, TANITA (Type BC-418MA, TANITA Corporation, Tokyo, Japan), and BIACORPUS (BIACORPUS RX 4000, Medical Healthcare GmbH, Karlsruhe, Germany) [20]. These data were based on a small study population, but still indicated that TANITA overestimated body fat percentage and fat mass and underestimated fat free mass compared to BIACORPUS [20]. In the present study, the main objective was to reevaluate these results and to specifically test in a larger study population whether the use of different devices for the determination of body composition in children and adolescents yields different results. For this purpose, we thus evaluated different devices and compared them with each other to obtain insight into the accuracy and reliability of the methods for the determination of body composition in patients with obesity during childhood and adolescence and to assess their usability in routine clinical practice.

Data on body composition assessments of children with obesity were obtained with TANITA and BIACORPUS. In addition, in order to expand this comparison and to obtain evidence as to which further comparison may be important for future research, these bioelectrical impedance-based data were compared to air displacement plethysmography BOD POD (BOD POD COSMED Inc., Concord, CA, USA) and DXA.

## 2. Materials and Methods

### 2.1. Patient Recruitment

Data from patients of three different studies performed at the Medical University Vienna were analyzed. All patients were recruited at the outpatient clinic of obesity, lipometabolic disorder and nutritional medicine at the Department of Pediatrics and Adolescent Medicine, Medical University of Vienna. The obesity outpatient clinic provides care by an interdisciplinary team of doctors, dieticians, nurses, psychologists and social workers.

Study #1, the BODCOP study—a retrospective study on data obtained between August 2015 and May 2016, included 67 children and adolescents who were patients at the above-named institution for whom body composition with TANITA and BIACORPUS, and anthropometric data were routinely assessed during their treatment schedule. All patients were aged under 18 years and had a body mass index (BMI) exceeding the 97th percentile according to the recommendations given by Kromeyer-Hauschild and colleagues [21]. The primary end point of this study was the comparison of both devices for measuring body composition in obese and non-obese individuals. The study was approved by the local Ethics Committee of the Medical University Vienna (EC Nr: 1357/2016).

Study #2, Children’s KNEEs study—a randomized controlled trial performed between September 2015 and May 2017, with data from 44 patients which were collected during a 12-week strength and neuromuscular exercise program for the lower extremity on knee load, pain and function in pediatric patients with obesity [22]. Patients were aged between 10 and 18 years and exceeded the 97th percentile described by Kromeyer-Hauschild [21]. The exclusion criteria were: (i) syndromes associated with obesity (e.g., Prader–Willi syndrome); (ii) chronic joint diseases; (iii) neuro-motor diseases; and (iv) any history of a lower extremity joint surgery. The bioelectrical impedance-based methods, TANITA and BIACORPUS, were performed in all patients. The primary end point of this study was to assess the impact of an exercise program for the lower extremity on knee load, pain and function in young patients with obesity. The study was approved by the local Ethics Committee of the Medical University Vienna (EC Nr: 1445/2013) and was registered at clinicaltrials.gov (accessed on 4 March 2021) NCT02545764. 

Study #3, the MotiMove Study—a pilot for an intervention study on movement in adolescents with obesity conducted between October and December 2019, included 12 patients aged between 14 and 18 years with a BMI exceeding the 90th percentile according to Kromeyer-Hauschild [21]. Despite the slightly different approach to include patients with a BMI above the 90th percentile, all patients in fact exceeded the 97th percentile as in the other two studies. The exclusion criteria were: (i) syndromes associated with obesity (e.g., Prader–Willi syndrome); (ii) chronic joint diseases; (iii) neuro-motor diseases; and (iv) any history of a lower extremity joint surgery. The primary end point of this study was to determine the effect of a physiotherapeutic group training on the motivation to perform physical activity in adolescents with obesity. In all patients, TANITA and BIACORPUS measurements were performed. In a subgroup, BOD POD and DXA were used to measure the body composition.

The Ethical Committee of the Medical University of Vienna approved the study (EC Nr: 1572/2019). In study #2 and #3, written informed consent was obtained from all parents and children.

### 2.2. Anthropometric Measurements

Anthropometric parameters which were recorded included body height and weight, and circumference at the waist, hip abdomen, and mid upper arm and were measured under the same standardized procedures in all patients. These latter measures of circumferences were not included in the present analysis. Procedures were as outlined previously [20], but a calibrated TANITA scale was used instead of a SECA scale (SECA 959, Seca Gmbh & Co., Hamburg, Germany). Body weight was measured in patients wearing light underwear, in standing position and within 0.1 kg precision. These values were also applied for the calculation of BIACORPUS-based body composition determination. Body height was measured in an upright facing position with feet together and the back against the wall to the nearest 0.1 cm as the maximum distance between the floor and the highest point on the head. Body weight and body height of each patient were assessed as a prerequisite to allow for the determination of the body composition with the methods used here.

### 2.3. Measurements of Body Composition

The present study applied four different methods for the assessment of body composition, two of these based on bioelectrical impedance analysis (TANITA, BIACORPUS), one on air displacement plethysmography (BOD POD), and one on dual X-ray absorptiometry (DXA). The main parameters determined and analyzed were body fat percentage (BFP, %), fat mass (FM, kg) and fat-free mass (FFM, kg). The two devices based on bioelectrical impedance analysis, TANITA and BIACORPUS, were performed in all patients. BOD POD and DXA were only conducted in patients from study #3. All body composition measurements were performed under the same standardized procedures. To reduce measurement variability, all measurements in each patient were conducted by the same trained team member, and under the same conditions such as overnight fast of the patient, with an empty bladder, wearing only light clothes, at the same daytime, and measurements were performed at room temperature by calibrated devices. 

### 2.4. TANITA and BIACORPUS

The assessment of body composition with the devices followed standard procedures as outlined before [20]. Patients were measured with the TANITA scale (Type BC-418MA, TANITA Corporation, Tokyo, Japan) and immediately afterwards with the BIACORPUS device (BIACORPUS RX 4000, Medical Healthcare GmbH, Karlsruhe, Germany), using a frequency setting of 50 kHz and applying 8 electrodes for both devices, assuring maximum comparability. Patients were measured in the morning, had fasted overnight and had emptied their bladder prior to the measurement.

### 2.5. BOD POD

The determination of body composition with air displacement plethysmography was conducted with the BOD POD Gold Standard Body Composition Tracking System (BOD POD COSMED Inc., Concord, CA, USA), following the instructions of the manufacturer. This type of measurement applies the principles of whole-body densitometry to determine body composition. For this, a high-precision weight assessment is combined with a body volume measurement and determination of the thoracic gas volume, and then, based on densitometric equations BFP, FM and FFM are calculated.

### 2.6. DXA

The DXA-based method for the determination of body composition applies the fact that fat and non-fat tissue attenuate X-rays to a different extent, allowing one to calculate the proportion of each compartment following a whole-body scan. 

Whole-body DXA was performed with a Hologic Horizon system A (Hologic Inc., Marlborough, MA, USA), applying the latest available Hologic Horizon A software release, for estimating body composition by the same trained technicians according to the prescriptions by the manufacturer and after daily calibration. Patients were scanned according to the most recent version of the official position for pediatric patients of the International Society for Clinical Densitometry (ISCD 2019). All patients were placed in supine position with the limbs in a standardized way according to the guidelines. Previous fractures or systemic diseases with a potential to influence the results had been excluded prior to the investigation and were excluded during reporting by checking the shape of the bones and the symmetry of the density values on the scout image. The precision and accuracy of DXA is reported as acceptable to define body composition in children [23]. Fat mass, lean tissue mass, and bone mineral content of the whole body were measured. The radiation dose, expressed as dose area product, was 7.9 cGy·cm^2^. 

### 2.7. Statistics

Statistical analyses were performed with the SPSS software package (SPSS Inc., Chicago, IL, version 24.0). Data were analyzed for normal distribution allowing the application of parametric tests. The results are expressed as means ± SD unless otherwise indicated. Assumptions were checked before conducting parametric tests. Data on body composition methods were analyzed with paired *t*-tests and a *p*-value of < 0.05 was considered as statistically significant. Further, data obtained with TANITA and BIACORPUS were compared by the Pearson correlation coefficient, and the Bland–Altman plot was used to assess the extent of agreement between the two methods [24]. The 95% limits of agreement were calculated as the mean difference ± 1.96 SD [25].

## 3. Results

### 3.1. General Characteristics of the Study Population

The data obtained from three independent studies were either separately evaluated for each study or presented as a pooled study population. As shown in Table 1, the common patient population consisted of 123 children and adolescents with 63% male participants and a mean age of 13.6 years. Study #3 included only adolescents aged 14 to 18 years. The overall BMI was 35.2 kg/m^2^ and slightly higher in study group 3 with an average BMI of 37 kg/m^2^.

### 3.2. Comparison of Bioelectric Impedance-Based Methods

Values on body composition determined with the bioelectrical impedance-based methods, TANITA and BIACORPUS are summarized in Table 2. In the overall study population with 123 patients, BFP amounted to 41.4% when determined with TANITA and 39.1% when assessed with the BIACORPUS device, a statistically highly significant difference (*p* < 0.001). Similar effects were shown for the FM with an average of 41.6 kg determined with TANITA and a significantly lower value of 38.6 kg determined with BIACORPUS (*p* < 0.001). In contrast, the average FFM of 56.2 kg seen with TANITA was significantly lower than that of 58.9 kg seen with BIACORPUS (*p* < 0.001). These differences were highly significant in patients from study #1 and #2, while in study #3 the differences were not significant, presumably due to the lower patient frequencies.

TANITA and BIACORPUS showed a significant difference in BFP (*p* < 0.01) and FFM (*p* < 0.001) between male and female participants, but not in FM (Table 3). As a result, BFP was lower in males compared to females. FFM was considerably higher in males than in females. Most of these differences were also observed and remained significant in study #1 and #2, except BFP in the TANITA of study #2, and in the BIACORPUS of study #3. The total FM measured with TANITA was higher in males than females, although this was not significant. In contrast, FM measured with BIACORPUS was equal in both genders and even slightly increased in females.

**Figure 1:**
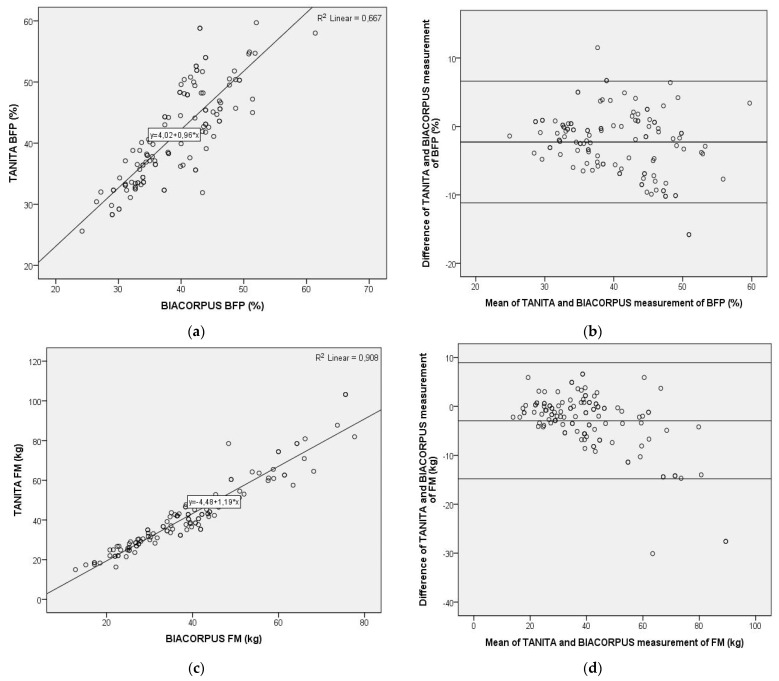

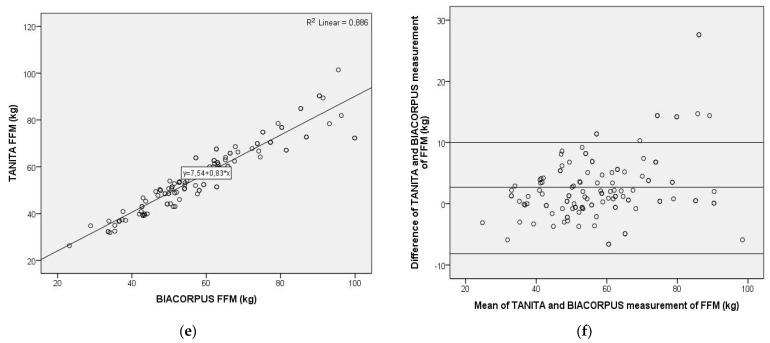
Pearson correlation analysis and Bland–Altman plot of body composition data obtained with two bioelectrical impedance-based methods, TANITA (Type BC-418MA, TANITA Corporation, Tokyo, Japan) and BIACORPUS (BIACORPUS RX 4000, Medical Healthcare GmbH, Karlsruhe, Germany). Left panel: Pearson correlation for body fat percentage (**a**), fat mass (**c**), and fat free mass (**e**), with R^2^ and the equation of the best linear fit given for each correlation plot. Right panel: Bland–Altman plots for body fat percentage (**b**), fat mass (**d**), fat free mass (**f**), with the middle line indicating the mean difference and the upper- and lower-line representing limits of agreement. BFP = body fat percentage, FM = fat mass, FFM = fat-free mass.

For further evaluation of differences in body composition determined with TANITA or BIACORPUS, Pearson correlation coefficients were used, and Bland–Altman plots generated. As shown in Figure 1, the Pearson correlation was highly significant between the TANITA and BIACORPUS-based determination of BFP (Pearson *r* = 0.817; *p* < 0.01, *n* = 123), of FM (Pearson *r* = 0.953; *p* < 0.01, *n* = 123), and of FFM (Pearson *r* = 0.941; *p* < 0.01, *n* = 123). This contrasts with the result obtained with the Bland–Altman plot, which displays the mean difference between the methods ± 1.96 SD. This analysis indicated important, potentially clinically relevant differences between both methods for BFP, FM and FFM. Thus, the average difference and the 95% limits of agreement was amounted to −2.27% (average) and from −11.17% to 6.62% (range) for BFP, to −2.93 kg (average) and from −14.81 kg to 8.95 kg (range) for FM, and to 2.69 kg (average) and from −8.17 kg to 13.56 kg (range) for FFM. The discrepancy between the methods increased at higher values of BFP, resulting in little agreement in the upper range of the data. Similar discrepancies were determined for FM, which differed by ± 12 kg at a mean of around 40 kg and displayed considerable skewness in the upper data range, and for FFM with a ± 11 kg range at a mean of around 57 kg.

### 3.3. Comparison of BOD POD- and DXA-Based Determination of Body Composition

In study #3, the body composition from 12 individuals was obtained with TANITA and BIACORPUS. In addition, seven patients were also subjected to comparative measurements using air displacement plethysmography and DXA-based assessments. In this sub-group, three persons were male (43%), the average age was 16.1 ± 1.5 years, and mean (±SD) values for height, weight and BMI were 166.9 ± 10.2 cm, 105.1 ± 17.3 kg, and 37.0 ± 4.7 kg/m^2^, respectively.

In spite of apparent differences, the most pronounced related to the determination of FM (ranging from 48.6 to 53.2 kg); due to the small sample size, we could not detect significant differences between methods (Table 4). Overall, there was a satisfying agreement for the BFP values, only BOD POD determined somewhat higher values, and a greater variability in for FM, which was lowest with BIACORPUS, and a reasonably good agreement for values on FFM.

## 4. Discussion

In line with a previous study [20], our current assessment, using a much larger sample size (*n* = 123 as compared to *n* = 38), confirmed that body composition determination in young patients with obesity yields significantly different results depending on whether it is conducted using the TANITA or the BIACORPUS device. The TANITA-based determination of body composition overestimated BFP and FFM relative to BIACORPUS, and significantly underestimated FFM. Pearson correlation indicated an expected association for all three parameters between both methods, and Bland–Altmann plot tests established clinically relevant differences. At an average BFP of around 40% measured with both methods, a difference of −2.3% may not seem excessive. However, these differences ranged from −11% to 6.6%, which appears rather large. In addition, we observed that the discrepancy between both methods increased at higher values of BFP. This showed considerable skewness for FM in the upper data range and was also unsatisfying for FFM. It seems obvious that body composition should be measured with the same bioelectrical impedance-based method. Hence, we recommend using exclusively the same BIA-device, either TANITA or BIACORPUS, for both clinical and research baseline and follow-up measurements of body composition within a study population group. This is supported by findings on adolescent patients with obesity [26] and on adult women with obesity, the latter study comparing TANITA with yet another device based on the same measuring principle [27]. Similarly, in a comparison of two overall rather similar bioelectrical impedance-based method with DXA in Taiwanese children, the impedance devices were found to overestimate FFM and to underestimate FM and BFP [16] and to agree with DXA to a different extent.

We could also confirm previously indicated gender-related differences in body composition [20]. Independent from the bioelectrical impedance-based method, male patients had significantly lower BFP and significantly higher FFM, whereas FM did not differ between genders. Physiologically, these differences may reflect the larger muscle mass in males and the enhanced fat deposition in developing females [28]. Overall, these data underline that gender differences need to be taken into account when comparing different groups [29]. 

For the same patient group, the four methods for body composition, TANITA, BIACORPUS, BOD POD and DXA showed a good agreement for BFP and FFM, but a higher variability for FM. It appears that TANITA and BIACORPUS, which are non-invasive and easily applied devices, could be appropriate to measure body composition in young patients with obesity. Previous studies found that bioelectrical impedance-based methods may be accurate enough to provide reliable measurements [30,31], and in other studies a more limited reliability was defined [26,32,33], except for individuals with obesity, but not for patients with morbid obesity [34], or a clear need of optimization for young patients with obesity [35], highlighting the need to be cautious when applying it. It thus appears important that future research conducts a thorough comparison of TANITA and BIACORPUS with DXA. As reported before, bioelectrical impedance-based methods may in some regards show excellent agreement with DXA but in others display considerable discrepancies [16,36]. It thus appears that for each specific device, a comparison is required to provide ultimate insight as to which bioelectrical impedance-based method is best comparable to the reference method. 

Some limitations have to be mentioned in interpreting our results. Most obviously, the sample size of the group with the BODPOD and DXA method comparison is rather small and therefore the results have to be considered as preliminary. An increased and consistent sample size in the comparison of all four body composition methods is the next step to apply a more robust methodology and to make sure that the outcomes are generalizable to the pediatric population affected by obesity. Another research focus should be especially the comparison of the different bioelectrical impedance devices with the gold standard DXA measurement to identify whether TANITA or BIACORPUS is more accurate in detecting the true body composition. The discrepancies between devices increases proportional to body fat percentage and therefore a follow-up study with lower body mass index percentile should be conducted to complement this study. 

A major strength of the study is that it fills a gap concerning different bioelectrical impedance analysis methods in pediatric individuals with obesity in a young western population. Other strengths are that the same devices, the same trained study team members as well as standardized procedures were used in all body composition measurements assuring that factors affecting measuring variability other than the devices applied were kept to a minimum. 

## 5. Conclusions

The evaluation of data from three independent studies in young patients with obesity could show that the body composition differed significantly for body fat percentage, fat mass and fat free mass depending on the bioelectrical impedance-based devices. TANITA and BIACORPUS, which are appropriate for clinical and research body composition measurements, cannot be used interchangeably and need to be strictly adhered to in order to receive reliable repetitive data. We recommend consistently using one of the easily applicable and non-invasive bioelectrical impedance-based devices, either TANITA or BIACORPUS, in pediatric patients with obesity. Further research on the comparison of the different bioelectrical impedance-based methods with DXA in young patients with obesity is warranted.

## Figures and Tables

**Table 1:** Demographic patient characteristics and anthropometric parameters.

**Characteristics**	**All Patients** **(*n* = 123)**	**Study 1 (*n* = 67)**	**Study 2 (*n* = 44)**	**Study 3 (*n* = 12)**
Male (absolute; %)	78 (63)	43 (64)	28 (64)	7 (58)
Age (years)	13.6 ± 2.6	13.4 ± 2.6	13.3 ± 2.4	15.9 ± 1.4
Height (cm)	165.2 ± 12.1	165.0 ± 12.7	164.2 ± 11.4	169.8 ± 10.4
Weight (kg)	97.5 ± 27.6	98.0 ± 28.7	94.0 ± 27.1	107.4 ± 21.8
BMI (kg/m^2^)	35.2 ± 7.0	35.4 ± 7.5	34.3 ± 6.8	37.0 ± 4.7

Results are presented as means ± SD or as numbers of subjects (%); BMI body mass index.

**Table 2:** Comparison of body composition parameters determined with bioelectrical impedance-based methods.

	All Patients (*n* = 123)			Study 1 (*n* = 67)			Study 2 (*n* = 44)			Study 3 (*n* = 12)		
	TANITA	BIACORPUS	*p*-Value	TANITA	BIACORPUS	*p*-Value	TANITA	BIACORPUS	*p*-Value	TANITA	BIACORPUS	*p*-Value
BFP (%)	41.4 ± 7.8	39.1 ± 6.7	0.000	41.6 ± 8.1	39.4 ± 6.8	0.000	40.7 ± 7.7	37.9 ± 6.0	0.000	43.0 ± 7.0	42.1 ± 8.2	0.481
FM (kg)	41.6 ± 17.8	38.6 ± 14.3	0.000	41.8 ± 18.5	39.0 ± 14.6	0.000	39.3 ± 17.4	36.3 ± 13.8	0.001	48.6 ± 14.5	45.2 ± 12.7	0.252
FFM (kg)	56.2 ± 14.2	58.9 ± 16.2	0.000	56.2 ± 14.3	59.1 ± 16.9	0.000	54.7 ± 13.6	57.7 ± 15.4	0.001	61.3 ± 15.9	62.1 ± 15.6	0.520

Results are presented as mean ± SD; comparison of the body composition methods was made using paired samples *t*-test; BFP body fat percentage, FM fat mass, FFM fat free mass. TANITA (Type BC-418MA, TANITA Corporation, Tokyo, Japan), BIACORPUS (BIACORPUS RX 4000, Medical Healthcare GmbH, Karlsruhe, Germany).

**Table 3:** Gender-specific values of body composition parameters determined with TANITA and BIACORPUS.

	All Patients (*n* = 123)			All Patients (*n* = 123)		
Parameter	Male (*n* = 78)	Female (*n* = 45)	*p*-value	Male (*n* = 78)	Female (*n* = 45)	*p*-value
		TANITA			BIACORPUS	
BFP (%)	39.9 ± 8.1	43.9 ± 6.9	0.007	36.5 ± 5.3	43.6 ± 6.6	0.000
FM (kg)	42.6 ± 18.8	39.8 ± 16.1	0.408	38.2 ± 13.7	39.4 ± 15.3	0.638
FFM (kg)	60.8 ± 14.3	48.1 ± 9.7	0.000	64.9 ± 16.0	48.5 ± 10.1	0.000
	Study 1 (*n* = 67)			Study 1 (*n* = 67)		
Parameter	Male (*n* = 43)	Female (*n* = 24)	*p*-value	Male (*n* = 43)	Female (*n* = 24)	*p*-value
		TANITA			BIACORPUS	
BFP (%)	39.9 ± 8.4	44.5 ± 6.8	0.024	36.8 ± 5.6	44.0 ± 6.2	0.000
FM (kg)	42.5 ± 19.4	40.5 ± 17.1	0.682	38.5 ± 14.1	39.9 ± 15.8	0.700
FFM (kg)	60.9 ± 13.9	48.8 ± 10.8	0.000	65.0 ± 16.7	48.5 ± 10.3	0.000
	Study 2 (*n* = 44)			Study 2 (*n* = 44)		
Parameter	Male (*n* = 28)	Female (*n* = 16)	*p*-value	Male (*n* = 28)	Female (*n* = 16)	*p*-value
		TANITA			BIACORPUS	
BFP (%)	39.9 ± 8.2	42.1 ± 6.6	0.362	36.8 ± 4.9	41.4 ± 6.3	0.002
FM (kg)	40.8 ± 18.8	36.6 ± 14.7	0.443	36.4 ± 13.8	36.0 ± 14.3	0.912
FFM (kg)	58.8 ± 14.0	47.5 ± 9.3	0.006	63.3 ± 15.5	48.1 ± 9.5	0.001
	Study 3 (*n* = 12)			Study 3 (*n* = 12)		
Parameter	Male (*n* = 7)	Female (*n* = 5)	*p*-value	Male (*n* = 7)	Female (*n* = 5)	*p*-value
		TANITA			BIACORPUS	
BFP (%)	40.6 ± 5.4	46.5 ± 8.0	0.158	37.7 ± 5.1	48.3 ± 8.0	0.017
FM (kg)	50.0 ± 15.0	46.5 ± 15.2	0.698	43.2 ± 11.7	48.1 ± 14.8	0.528
FFM (kg)	68.2 ± 17.6	51.6 ± 5.5	0.072	70.8 ± 14.0	50.0 ± 7.8	0.014

Results are presented as mean ± SD; comparison of the body composition methods was made using independent samples *t*-test; BFP, body fat percentage, FM, fat mass, FFM, fat free mass.

**Table 4:** Comparison of body composition data obtained with TANITA, BIACORPUS, BOD POD and DXA.

Parameter	TANITA	BIACORPUS	BOD POD	DXA
BFP (%)	46.3 ± 7.3	46.0 ± 8.0	48.5 ± 4.8	46.8 ± 3.4
FM (kg)	53.2 ± 16.3	48.6 ± 12.1	50.6 ± 8.0	51.5 ± 7.7
FFM (kg)	56.1 ± 11.2	56.6 ± 12.5	54.5 ± 12.2	56.6 ± 10.7

Results are presented as mean ± SD, *n* = 7; DXA: dual-energy X-ray absorptiometry, BFP: body fat percentage; FM: fat mass; FFM: fat free mass.

## Data Availability

Data and intervention materials are available upon request to the corresponding author.

## Abbreviations

BFP: Body fat percentage; BIA: Bioelectrical impedance analysis; BMI: Body mass index; DXA: Dual energy X-ray absorptiometry; FFM: Fat free mass; FM: Fat mass
